# Successful instillation of professionalism in our future doctors

**DOI:** 10.15694/mep.2021.000173.1

**Published:** 2021-06-14

**Authors:** Edward Wargent, Claire Stocker

**Affiliations:** 1 University of Buckingham; 2 Aston University

**Keywords:** Professionalism, communities of practice, professional values, medical students

## Abstract

This article was migrated. The article was marked as recommended.

Dynamic approaches are required in teaching professionalism to medical students. Awareness of this issue has both arisen from and generated by a dramatic increase in publications relating to professionalism teaching in medical education. This report explores the current state of defining professionalism and shows that current literature reveals a strong proclivity to adopting “Communities of Practice” as the learning paradigm most likely to successfully instil professional values. This pedagogy is then critiqued with regards to the requirement of an undergraduate curriculum with the conclusion that Communities of Practice should be pertinent to successfully empowering medical students’ professionalism.

## Introduction

Professional values are an essential component of Good Medical Practice ( General Medical Council, 2019). However, the teaching of professionalism has traditionally resided within the “hidden curriculum” ( Jackson, 1968). In 2007, a review of how professionalism is acquired by medical professionals and students concluded that it was “imperative for medical education to instil and foster professionalism” and that there was a need for health care regulators to institutionally define professionalism ( Hilton and Southgate, 2007). This becomes particularly relevant with the recent proposal that leaders of the community of physicians have a responsibility, under Medicine’s “Social Contract” to “ensure the maintenance of medicine’s professional status” (Cruess and Cruess, 2020). This report explores the progress in defining what is meant by professionalism and then evaluates the literature on teaching professionalism within current pedagogical theory with an aim to identifying, and then critiquing, an educational paradigm that may best be adopted to instil professionalism in an undergraduate curriculum.

## Defining professionalism

Dictionaries define professionalism along the lines of the conduct, aims, or qualities that characterize or mark a profession or a professional person; or the following of a profession for gain or livelihood ( "professionalism" Merriam-Webster, 2017). Most prominent of these for most people is likely conduct, as the journalist Alastair Cooke said, “a professional is someone who can do his best work when he doesn’t feel like it” (quote attributed to Cooke in Medical Protection Guide, Medical Protection Society, 2015a), implying that true professionalism is more than a question of payment. This was nicely summed up by Dame Janet Smith when she defined professionalism as “a basket of qualities that enables us to trust our advisors” for a Royal College of Physicians working party ( Working Party of the Royal College of Physicians, 2005). The report concluded that professionalism for doctors is a set of values, behaviours, and relationships that underpins the trust the public has in doctors.

In a way, professionalism is at the heart of the General Medical Council (GMC) as it was founded in 1858 to stop charlatans and “quacks” practicing and to ensure uniformity of medical education (Medical Protection Guide, Medical Protection Society, 2015b). In 2002 (and revised in 2013 and amended in 2019) the GMC published Good Medical Practice which details the standards of care and conduct expected to be upheld by all medical practitioners ( Table 1).

**Table 1:** Summary of the GMC’s expectations of professionalism categorized into the five most common areas of fitness to practice cases.

Professionalism expectation	Duty to patients	Duty to colleagues
Probity	Patients confide personal information and must trust the doctor-patient relationship completely.	Records must be complete, accurate, and contemporary. This applies universally, not only to medical reports, e.g. in dealing with complaints and investigations, both internal and external.
Clinical Care	Appropriate care depends not only on knowledge and competency but also on diligence to records.	Professional medics understand the importance of seeking and accepting assistance.
Relationships	Communication, politeness, and respect are vitally important for doctor-patient relationships.	Teamwork is vital to the proper delivery of care. A true professional in healthcare respects colleagues and proactively communicates and responds to deficiencies.
Social media	When referring to professional matters on social media the above values must be respected just as in the clinic.

Adapted from Professionalism: A Medical Protection Guide ( Medical Protection Society, 2015c).

## Teaching professionalism

**Figure 1.**
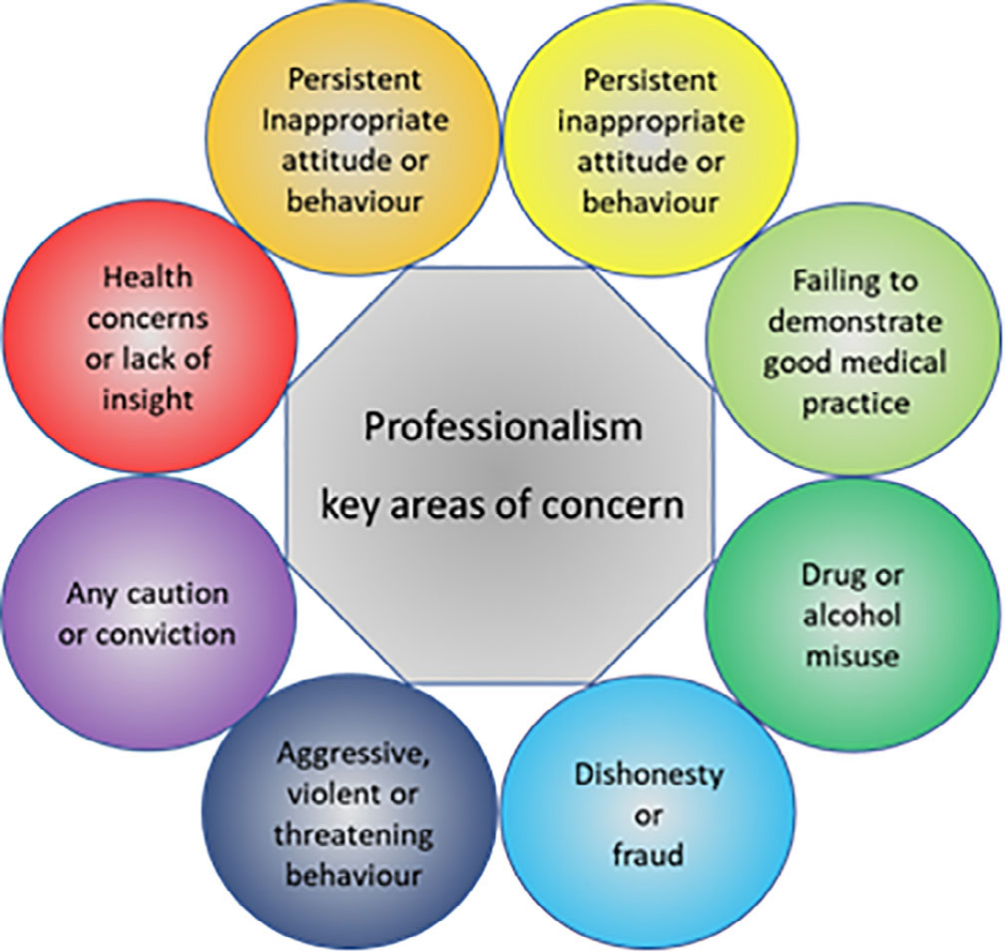
Core values for achieving good medical practice: guidance for medical students.

Adapted from Medical students: professional values and fitness to practice ( General Medical Council, 2016).

Teaching professionalism raises questions of how to make the students aware and understand their expectations, provide them with experiences, and assess the outcomes ( Stern and Papadakis, 2006; Archer *et al.*, 2008). It has been postulated that a formal integration may not be possible and that it is the environment of the teaching institution itself that must reflect and teach the qualities of professionalism ( Cruess, 2006) and traditionally the values of professionalism have been within the realm of the “hidden curriculum”. This presents a major stumbling block - the teaching of professionalism is ill-defined, yet medical students are expected to meet professional standards of professionalism (summarized in Figure 1, General Medical Council, 2016). Many medical students arrive straight from school and the step-up from pupil to professional within a short time scale ought to, in fairness, be facilitated by an organized approach.

**Figure 2.**
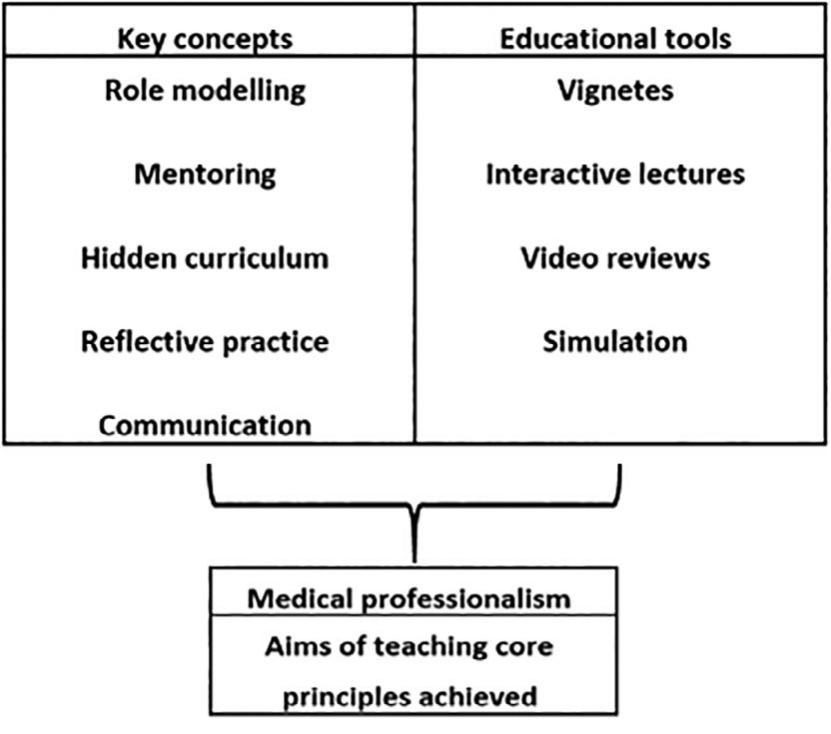
Concepts and tools commonly employed in teaching professionalism.

The number of publications referring to the art of teaching professionalism in medical education has increased dramatically in recent years (Supplementary Table 1), highlighting the growing awareness of the importance of tackling how professionalism is taught and assessed. In 2004 the Accreditation Council for Graduate Medical Education ( ACGME, 2004) published a report that recommended the teaching of professionalism, the outcomes ( Figure 2) since being adopted by many US medical schools ( Guraya, Guraya and Almaramhy, 2016). A later study of US and Canadian medical schools found that only 50% had any professionalism training and less than 8% tailored the need according to individual student’s needs ( Lown *et al.*, 2007) and recommended development of models designed to assess how to teach professionalism. Similarly, educators in 18 of 23 UK medical schools thought that an over-reliance on the “hidden curriculum” was undermining professionalism training and the area required improvement ( Stephenson, Adshead and Higgs, 2006). Upon examination of 43 studies that followed two themes are consistently appraised as being the most effective when teaching professionalism: role-modelling and personal reflections ( Birden *et al.*, 2013).

Summarized from ACGME recommendations ( Ludwig, 2014).

### Role-models

Practitioners experiences and observations have long been recognized as important in developing professionalism ( Wesley and Buysse, 2001), with some studies suggesting that it is the actions and attitudes students see in role-models that most affect their own professional behaviours ( Inui, 2003; Levenson, Atkinson and Shepherd, 2010). What students pick up fastest from role-models are the behaviours and attitudes that seem to them successful considering their own goals and experiences so that role-modeling is influenced by what students want to learn ( Bandura, 1986). However, students tend to have an incomplete vision of what professionalism means and focus on core competencies so when a role-model appears to value clinical skills and knowledge above professionalism this belief gets reinforced ( Goldie *et al.*, 2007).

The use of role-modelling to teach professionalism has traditionally been unregulated within the “hidden curriculum”, and as such could succumb to these pitfalls and be detrimental to professional development ( Goldie *et al.*, 2007). Therefore, whilst role-modelling is important a more organized approach than the “hidden curriculum” is necessary ( Cruess and Cruess, 2006), ideally complemented by reflection on observed behaviour ( Goldie *et al.*, 2007).

### Reflection

In concordance with the literature, direct experience of professionalism teaching in medical education shows that critical reflection is fundamental ( Goldie *et al.*, 2007). In medical practice/studies there are four areas of reflection: 1. Technical examination of skills and competencies. 2. Professional performance to patients. 3. Exploration of alternate ways to solve problems. 4. Peer professionalism ( Hatton and Smith, 1995). The object of all four is to apply the reflection in practice - “reflection in action”. ( Schon, 1987). The act of reflection itself can take two forms: 1. Learning organisations. And 2. Communities of Practice.

Promoted by Peter Senge ( Senge, 1990), a learning organisation is a means to enhance an organisation through individual self-development, learning and reflection ( Starkey, 1996). As such individual experiences and thoughts are directed upwards in the organisation hierarchy to be disseminated downwards again essentially via Standard Operating Procedures and, whilst professional behaviour is a requirement, its purpose is to facilitate the system, not promote a professional attitude in the conduct of practice ( Wesley and Buysse, 2001).

Communities of Practice (CoPs) are groups of people that share a common interest or passion and through social interaction enhance their knowledge and learn how to be better practitioners ( Lave and Wenger, 1991). The social interaction is key and provides opportunities for collaborative reflection to develop skills and identity ( Rogoff, 1994; Westheimer and Kahne, 1993). Peter Senge himself concludes that Communities of Practice are better than learning organisations for learning professional behaviour and attitude ( Senge, 1990).

## Choosing a learning theory to teach professionalism

**Figure 3.**
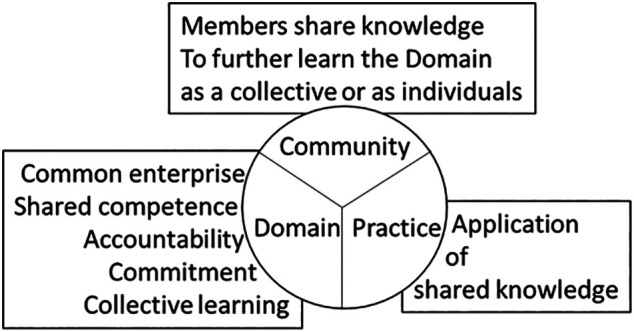
The three elements of Communities of Practice.

Coupling the key terms for professionalism training, “professionalism”, “reflection” and “role-models/modeling”, with the 31 learning theories described by Millwood ( Millwood, 2013) in a PubMed search indicates which may be the most appropriate to the teaching of professionalism, and Figure 3 summarizes the results with the theories that returned publications for all three (full results shown in Supplementary Table 1). The learning theory that has been the focus of most academic research in this area is experiential learning - the process whereby knowledge is created through the transformation of experience ( Kolb, 1984). Integrating reflection within the curriculum improves engagement and is key to experiential learning ( Gostelow and Gilshen, 2017). The key to which form the reflection should take is given with the learning theory second on the list - Communities of Practice.

Whilst reflecting on learning core material and skills is important, it is in the relating of these competencies to people that the true nature of the work can be realised ( Wesley and Buysse, 2001) and a truly reflective practitioner takes reflection beyond personal adjustment to collective inquiry involving roles and values in a social context ( Bullough and Gitlin, 1991). The acquisition of competence in professionalism is developed along a continuum ( Leach, 2004), implying that the greater the experience pool on which to reflect the greater the personal enhancement of professionalism. These observations suggest that it is the reflection of experiential learning within CoPs that the nature of professionalism is best learned, and it is through continuous reflection of experiences that students develop their professional identity ( Liljedahl *et al.*, 2016). The role-modelling in CoPs with practitioners as members is a sort of mentoring ( Mann and Gaufberg, 2016) and this is important as direct mentorship is recognized as having greater effect in developing good disposition and commitment than observing role-models ( Taylor, Taylor and Stoller, 2009).

Regarding the other learning theories in Table 2, situated learning returned several results, but again this indicates utilizing CoPs as they are a form of situated learning. Most of the results for experiential education referred to experiential learning and the two are often mistakenly used interchangeably ( Mughal and Zafar, 2011). Papers, particularly the reviews ( Rose, Rukstalis and Schuckit, 2005; Johnston, 2006; Mellis, 2008) for “learning styles” contained useful ideas for mentors rather than course construction and so will not be discussed here but will be kept in mind considering that multiple learning theories can be incorporated into CoPs ( Cruess, Cruess and Steinert, 2018). The results for educational objectives referred to the literal phrase, not the learning theory and constructivism is already extensively employed in medical education ( Dennick, 2016) and while it provides a foundation for the building of CoPs ( Hutchings, Scammell and Quinney, 2013) current practice has not satisfactorily addressed the professionalism issue.

See Supplementary Table 2 for the search results for all 31 learning theories described by Millwood ( Millwood, 2013).

**Table 2:** Learning theories and paradigms that gave search results for articles in PubMed when linking the theory with the terms “professionalism”, “reflection” and “role-models/modelling”.

Learning theory/paradigm	Professionalism	Reflection	Role-models (role modelling)
Experiential Learning	53	197	24
Communities of Practice	12	27	9
Situated Learning	9	23	3
Learning Styles	7	19	6
Experiential Education	8	26	2
Educational Objectives	7	9	5
Constructivism	3	21	2

## Communities of Practice

Developed from the theory of situated learning, communities of practice are ways of facilitating the spreading of knowledge through social interaction with an aim of enhancing the practice of all members ( Lave and Wenger, 1991). Through social reflection, the components of CoPs ( Figure 3) collectively facilitate the contemplation of peer and role-model experiences to aid individuals develop skills and a personal identity within the practice ( Wenger, 1998). As such they are thought to be particularly suited to the development of professionalism and professional identity ( Senge, 1990; Woods, Cashin and Stockhausen, 2016), which is so integral to becoming a competent health care professional ( Plack and Driscoll, 2017). Because CoPs are self-regulated concerns have been raised as to whether CoPs can be incorporated into an organisation where the learning required is governed by objectives and therefore formally structured ( Vann and Bowker, 2001). Demurrals focus on the inherent nature of commercial organisations that may foster unprofessional attitudes ( Lee, Parslow and Julien, 2002; Eraut, 2002; Kerno, 2008; Gourlay, 1999) and as such may not apply to university educational programs, which may be well suited to supporting CoPs ( Cox, 2005).

## Integrating CoPs into professionalism tutoring

Incorporating CoPs into the reflective processes of a Med Ed curriculum requires a constructivist approach to learning to allow the students to actively engage in their own education ( Hutchings, Scammell and Quinney, 2013). With this proviso in operation, three points have been suggested to be important for successful implementation of CoPs regarding participants benefaction: 1. members should bring experience from diverse resources; 2. members should understand the CoP’s purpose and requirements; 3. one all-encompassing CoP works better than several CoPs isolating different aspects of the topic ( Palincsar *et al.*, 1998).

Regarding point 1, it has been proposed that for a student’s education to be truly patient-centred, clinicians should be the sole source of role-models as it is they who are the experts in doctor-patient and student-patient relationships not the patients or students as they alone have the experience to understand the nuances of the relations ( Bleakley and Bligh, 2008). It has been observed that during experiential learning of consultant sessions, students and patients take a submissive role and it is the doctors ability to promote student-patient interaction that underlies the learning of professional expertise ( Ashley *et al.*, 2009). Others suggest that patients make good clinical educators ( Towle and Godolphin, 2015), particularly as part of group inter-professional learning ( Towle and Godolphin, 2013). There is a two-way social contract between doctors and patients and the upholding of this contract defines professionalism ( Cruess, 2006) and so the patient’s view of their treatment is just as important as the doctor’s and should be considered in social reflections on professionalism.

There are challenges to overcome when incorporating CoPs into an educational program ( Wesley and Buysse, 2001) and these can be applied to the Med Ed program:

1. *Making reflection a shared value.* Resistance to reflective practice can occur when its importance is undervalued ( Hatton and Smith, 1995). Some students may see reflection/CoP participation as a distraction from learning clinical theory and competencies. The danger of individual reflection is that discrepancies between GMC/institution ideals and workplace realities may not be addressed, resulting in an incomplete understanding of the professionalism issue being considered. Practitioners and staff not used to situated learning styles may need orienteering to comprehend the opportunities they offer, but it is necessary for the faculty to relinquish control for the CoP to function (Bullogh and Gitlin, 1991).
2. *Incorporating a CoP into the Med Ed program.* The distinction between pre-clinical and clinical learning is blurring ( McNelis *et al.*, 2011) providing more meaningful experiences of how to conduct oneself and providing the core members of a CoP that are directly involved in the experiential learning ( Goldie *et al*., 2015). Schools that start clinical placements in year 1 may have an advantage in this regard.
3. *Sustaining CoPs over time.* This involves motivating and obtaining commitments from the members. Multiple incentives sustain solutions that benefit all members, including 1. Cross-fertilization of ideas, 2. Solutions, Shared leadership in the dissemination of knowledgeable experience between generations of doctors (and patients), 4. Understanding multidisciplinary professionalism, 5. The sense of making a difference. The inclusion of patients represents a shift from working on to working with the community ( Waddock, 1999). This may require reframing professional relationships and involve increased public awareness via community outreach programs.

## Formal versus informal professionalism tutoring

Theoretically, CoPs may be a good way to teach professionalism, and by being mindful of the challenges of implementing them it appears possible to do so. But like role-modelling via the “hidden curriculum” CoPs are an informal method of teaching and so can we be sure they do not have the same disadvantages?

There are two published reports of medical schools that purport to have incorporated CoPs with the specific aim of teaching professionalism - Keck School of Medicine at the University of Southern California which was purely descriptive of their methods ( Elliott *et al.*, 2009) and a more analytical study by the University of Glasgow ( Goldie *et al*., 2007). Both institutes employed project teams of 6-8 pre-clinical students supported by clinical facilitators rather than true CoPs with course co-ordinators having the final say in topics for discussion: as such none of the requirements for CoPs are satisfied ( Palincsar *et al*., 1998).

Educators reported that the students tended to think about what they need to know to pass their exams, not what they need to know to be a doctor, resulting in them esteeming problem-based learning over professionalism training ( Goldie *et al*., 2007). So, despite educator-facilitated guidance through the GMC requirements and case-studies students were failing to comprehend and assimilate the professionalism concept. Students were motivated by guest role-models as they provided an opportunity for joint reflection, which stimulated interest in all aspects of professionalism not just the desire to integrate professionalism with clinical skills. This was equally true when the role-models were expert patients as well as clinicians. Due to the format, these had to take place as whole-class discussions as the guests could not facilitate everyone in the group format and so, for a short time, the reflection on professionalism took a step closer to a true CoP and this was more effective at teaching professionalism than the project teams ( Goldie *et al*., 2007).

## Assessment

Both the Glasgow and USC courses assessed professionalism learning principally by records in the students’ portfolios (aside from course attendance and completion), but this was felt to be ineffective because portfolios completion did not count towards an exam grade they were undervalued, with many students leaving the completion of the portfolio until the end of the year, reducing the effectiveness of the reflection. Tutors felt that 1-1 feedback was better at instilling professionalism (but were unfortunately under-resourced in terms of time to officially do that). This concurs with a study by Papadakis who concluded it is feedback from assessment that improves professional behaviour rather than simply learning professionalism for assessment itself ( Papadakis, Loeser and Healy, 2001).

The portfolio is meant as a record of personal development. It is uncollaborative and when used for reflection is more suited to learning organisations than communities of practice. Whilst it may or may not be a good assessment tool, it should be a good means of recording and monitoring assessment and the argument that it may not be because students may neglect it is not a good one, because portfolio completion is a GMC requirement and failing to do so is itself a professionalism issue. The portfolio then becomes the means of recording professionalism development, but the question then arises if CoPs can be the means of assessment as well as teaching?

CoP effectiveness is positively correlated with diversity of members and as such should include course staff members as well as community practitioners and the students themselves. Learning professionalism is most effective by feedback as this not only assesses but also enhances the learning of professionalism. Immediate feedback from will occur naturally as part of CoP discussions, and could be recorded by educators, but it would be more efficiently recorded by the students themselves, and this would be in keeping with the constructivist learning style preferred by UoB. Students can reliably evaluate their peers ( Arnold *et al*., 1981) and peer-rating forms have high inter-assessor concordance when measuring professional behaviour ( Davis and Inamdar, 1988). So not only is this feasible it is also especially desirable for professionalism training considering that peer-assessment facilitates the professional attributes of self-regulation and accountability ( Leach, 2002).

## Conclusions

**Figure 4.**
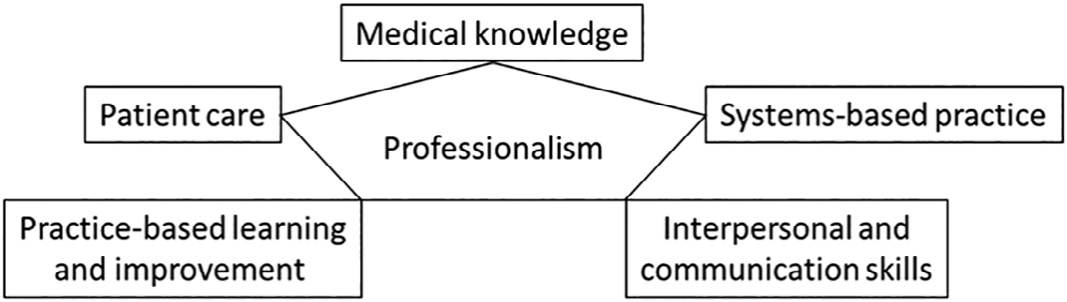
Six core values of teaching professionalism.

There is a need for greater devotion to the development of professionalism in medical students than is currently adopted. Analysis of professionalism teaching currently focuses on the use of direct teaching, experiential learning, or combining the two - the latter being preferential ( Cruess and Cruess, 2006; Steinart *et al.*, 2007). The awareness of professional responsibilities and their importance may well best be raised by the observation and mentoring of positive role-models supported by social reflection with peers and practitioners within CoPs. Through this reflection on experiential learning or other case studies students develop their own professional identity and become competent professionals deserving of patients’ trust and enabling them to value and assess their peers’ professionalism ( Cruess, Cruess and Steinert, 2019). For whilst experiential learning is important in medical education, it is but one facet of many - all of which are tied together by the understanding and adoption of the professionalism concept ( Figure 4). These facets form the backbone of a definition of professionalism that conforms with the six domains of professionalism required for defining professionalism ( Hilton and Southgate, 2007). The GMC has similarly adopted a backbone of prime requisites that define professionalism as summarized in Figure 1 ( General Medical Council, 2016; Medical Protection Society, 2015c).

Summarized from ACGME recommendations ( Ludwig, 2014).

The Hippocratic Oath states not only that doctors gain their patients’ trust and honestly act in their best interests, but that they also acknowledge the effort that other doctors put in to train them by passing on their own knowledge and experience to the next generation of doctors (quote attributed to Hippocrates in The Hippocratic Oath, translated by Ludwig Edelstein, 1943). Incorporation of CoPs into medical education creates an extra dimension for the professional community to contribute to medical education to the benefit of doctors, students, and educators alike. CoPs have been shown to be mutually beneficial for all participants, including learners and educators regardless of age and experience, within a school environment ( Mitra, 2008).

Clinical exposure represents the best source of topics for reflection and yet most schools, including the examples from Glasgow and USC have professionalism training only in the pre-clinical phase. Considering this and the tendency for students to focus on clinical skills over professionalism increases once they enter the clinic, the two ought to occur simultaneously - especially so as professionalism and clinical experiential learning enhance each other ( Pfeiffer, Chen and Tsai, 2016). Such a practice would facilitate the assimilation of CoPs within the educational environment.

The fact that professionalism is generally scantly incorporated into core curricula and that the teaching methods vary greatly makes the teaching of professionalism different in each institution. Designing a scaffold for professionalism training therefore starts from different foundations for every medical school and so must be designed specifically for each medical education programme ( Cruess, 2006). Whilst there is no “best” way to achieve this, the critical factors in all successful strategies have been laid down, and by examining the work of others the adoption of effective CoPs into undergraduate medical education curricula ought to be both feasible and rewarding in the successful instillation of professionalism in our future doctors.

## Take Home Messages

• Professionalism is becoming increasingly well-defined by medical education regulatory bodies.
• Communities of Practice ought to be well suited to the teaching of professionalism to medical students in undergraduate curricula.

## Notes On Contributors

**Claire Stocker** is Phase 1 Lead at Aston University Medical School, Birmingham, UK. Prior to that, she was Educator and Learner Support Leads at the University of Buckingham Medical School, Buckingham, UK. ORCiD: https://orcid.org/0000-0002-0178-7349

**Edward Wargent** is a Research Fellow and Biomedical Lecturer in the Institute of Translational Medicine, University of Buckingham, Buckingham, UK. He specializes in Obesity and Diabetes Research and Drug Development. ORCiD: https://orcid.org/0000-0002-8545-2430
