# Supplementary material for: Successful instillation of professionalism in our future doctors
Source: MedEdPublish (2016). 2021 Jun 14;10:173. [Version 1] doi: 10.15694/mep.2021.000173.1 (PMC10939588; doi:10.15694/mep.2021.000173.1)
Supplement: Peer review history

# Reviewer report: Reviewer response for version 1

**DOI:** 10.21956/mep.18957.r27134

**Published:** 2021-10-06

**Authors:** Trevor Gibbs

**Affiliations:** 1 AMEE

This review has been migrated. The reviewer awarded 4 stars out of 5 An interesting and well structured paper that provides more insight into a difficult topic. Initially I thought I was going to read a systemmatic review of the subject , but I soon realised that this was not the case. The paper presents a relatively new approach to the subject of developing professionalism; the case was presented well and will be very useful for all those involved in the subject.I did find it somewhat "advanced" in its approach and wonder how useful it would be to those faculty new to the subject and from less well resourced countries where health professions education is still developing. For that reason I gave the paper 4 stars rather than 5

Reviewer Expertise:

NA

No decision status is available

# Reviewer report: Reviewer response for version 1

**DOI:** 10.21956/mep.18957.r27135

**Published:** 2021-07-09

**Authors:** Richard Hays

**Affiliations:** 1 James Cook University

This review has been migrated. The reviewer awarded 4 stars out of 5 This article is a nice, compact overview of the key issues in the shaping of professionalism in medical education. Although rather UK/GMC-centric, much of the content is relevant elsewhere, although there are some differences in professional norms between nations and cultures. The important message that comes through strongly is that professionalism needs to be a deliberate curriculum component that should be delivered with several learning strategies and assessed formally. Appropriate references are provided for readers to explore specific issues more comprehensively. I recommend this to all in medical educators, particularly in revising or developing new programs.

Reviewer Expertise:

NA

No decision status is available
